# Dynamic Mechanical Thermal Analysis Data of Sheets Made from Wood-Based Cellulose Fibers Partially Converted to Dialcohol Cellulose

**DOI:** 10.1016/j.dib.2017.07.014

**Published:** 2017-07-14

**Authors:** Eric Linvill, Per Larsson, Sören Östlund

**Affiliations:** a KTH Royal Institute of Technology, Department of Solid Mechanics, SE – 100 44 Stockholm; b KTH Royal Institute of Technology, Department of Fibre and Polymer Technology, SE – 100 44 Stockholm; c BiMaC Innovation, KTH Royal Institute of Technology, SE – 100 44 Stockholm

## Abstract

This data article contains the dynamic mechanical thermal analysis (DMTA) results for sheets made from cellulose fibers partially converted to dialcohol cellulose as presented in “Advanced Three-Dimensional Paper Structures: Mechanical Characterization and Forming of Sheets Made from Modified Cellulose Fibers” by Linvill et al. [1]. See Larsson and Wågberg [2] for a description and characterization of the material as well as how the material is produced. The DMTA tests were conducted at four different relative humidity levels: 0, 50, 60, and 70% RH, and the temperature was swept between 10 and 113 °C. The DMTA results enable the understanding of the elastic, viscoelastic, and viscoplastic mechanical properties of this material at a wide range of temperature and relative humidity combinations.

**Specifications Table**

**Table:** 

Subject area	Solid Mechanics, Material Science, Chemistry
More specific subject area	Characterization of Materials, Rheology
Type of data	Raw data with summary graphs
How data was acquired	Q800 DMTA from TA Instruments with a moisture- and temperature-controlled chamber
Data format	Raw
Experimental factors	Samples were kept at 23 °C and 50 % RH between production of the material and DMTA testing
Experimental features	Samples were kept in a moisture- and temperature-controlled chamber and then subjected to a cyclic, uniaxial load. During these cycles, the temperature was swept from 10 to 113 °C and back again multiple times. During these sweeps, the humidity was kept approximately constant via a control system.
Data source location	Innventia, Stockholm, Sweden
Data accessibility	Data not held within a public repository at the following link:http://urn.kb.se/resolve?urn=urn:nbn:se:kth:diva-209252

**Value of the data**

• A similar dataset for sheets made from cellulose fibers partially converted to dialcohol cellulose has never been made public before.
• This data creates understanding of the elastic, viscoelastic, and viscoplastic mechanical properties of this material.
• The glass transition temperature of this material at various humidity conditions can be directly postulated from the dataset.

## 1 Data

The raw data, summary graphs of the raw data, and a guide describing the variables and each specimen's dimensions are contained within the file: SupportingInformation_DMTA.xlsx, which can be downloaded from the link: http://urn.kb.se/resolve?urn=urn:nbn:se:kth:diva-209252

This raw data contains the dynamic mechanical thermal analysis (DMTA) results for sheets made from cellulose fibers partially converted to dialcohol cellulose as presented in “Advanced Three-Dimensional Paper Structures: Mechanical Characterization and Forming of Sheets Made from Modified Cellulose Fibers” by Linvill et al. [1]. A description and characterization of the material as well as how the material is produced is presented by Larsson and Wågberg [2]. As typical to dynamic mechanical thermal analysis (DMTA), the storage modulus, loss modulus, and tangent delta are the main outputs. Additionally, other states are measured, such as the elapsed time, the temperature within the test changer, the displacement wave amplitude, the strain wave amplitude, the change of the displacement wave mean, the crosshead location, the distance between the clamps at zero force, the measured relative humidity just above the sample's surface and of the test chamber, as well as the in-flow of dry and moist air.

## 2 Experimental Design, Materials and Methods

All samples were conditioned at 23 °C and 50% relative humidity (RH) between the production of the paper and the DMTA testing. In other words, the specimens were subjected to no significant cyclic/changing temperatures or relative humidity levels prior to testing.

The DMTA was conducted at four separate times at four set relative humidity levels: 0, 50, 60, and 70% RH. During each DMTA run, the desired relative humidity and a temperature of 10 °C was achieved and maintained for the first 120 minutes. The temperature was then swept from 10 to 113 °C for 206 minutes, held constant at 113 °C for 30 minutes, swept from 113 to 10 °C for 206 minutes, and then held at 10 °C for 30 minutes. The temperature sweep cycle described in the previous sentence was conducted a total of three times or until the sample failed.

## References

[1] Linvill E., Larsson P., Östlund S. (2017). Advanced Three-Dimensional Paper Structures: Mechanical Characterization and Forming of Sheets Made from Modified Cellulose Fibers. Materials & Design.

[2] Larsson P.A., Wågberg L. (2016). Towards natural-fibre-based thermoplastic films produced by conventional papermaking. Green Chemistry..

