# Supplementary figures and images for: Dynamic Mechanical Thermal Analysis Data of Sheets Made from Wood-Based Cellulose Fibers Partially Converted to Dialcohol Cellulose
Source: Data Brief. 2017 Jul 14;14:504–6. doi: 10.1016/j.dib.2017.07.014 (PMC5562104; doi:10.1016/j.dib.2017.07.014)

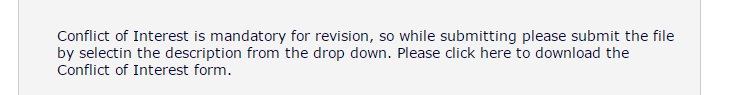


I can’t “click here”, because there is no hyperlink.

Supplement: Supplementary file 1 — Supplementary material [file mmc1.docx]
